# Functionalized Silver and Gold Nanomaterials with Diagnostic and Therapeutic Applications

**DOI:** 10.3390/pharmaceutics14102182

**Published:** 2022-10-13

**Authors:** Navid Rabiee, Sepideh Ahmadi, Siavash Iravani, Rajender S. Varma

**Affiliations:** 1 School of Engineering, Macquarie University, Sydney, NSW 2109, Australia; 2 Department of Materials Science and Engineering, Pohang University of Science and Technology (POSTECH), 77 Cheongam-ro, Nam-gu, Pohang 37673, Gyeongbuk, Korea; 3 Department of Medical Biotechnology, School of Advanced Technologies in Medicine, Shahid Beheshti University of Medical Sciences, Tehran 19857-17443, Iran; 4 Cellular and Molecular Biology Research Center, Shahid Beheshti University of Medical Sciences, Tehran 19857-17443, Iran; 5 Faculty of Pharmacy and Pharmaceutical Sciences, Isfahan University of Medical Sciences, Isfahan 81746-73461, Iran; 6 Regional Centre of Advanced Technologies and Materials, Czech Advanced Technology and Research Institute, Palacký University in Olomouc, Šlechtitelů 27, 783 71 Olomouc, Czech Republic

**Keywords:** functionalization, gold nanomaterials, silver nanomaterials, drug delivery, cancer theranostics, biosensing

## Abstract

The functionalization of nanomaterials with suitable capping ligands or bioactive agents is an interesting strategy in designing nanosystems with suitable applicability and biocompatibility; the physicochemical and biological properties of these nanomaterials can be highly improved for biomedical applications. In this context, numerous explorations have been conducted in the functionalization of silver (Ag) and gold (Au) nanomaterials using suitable functional groups or agents to design nanosystems with unique physicochemical properties such as excellent biosensing capabilities, biocompatibility, targeting features, and multifunctionality for biomedical purposes. Future studies should be undertaken for designing novel functionalization tactics to improve the properties of Au- and Ag-based nanosystems and reduce their toxicity. The possible release of cytotoxic radicals or ions, the internalization of nanomaterials, the alteration of cellular signaling pathways, the translocation of these nanomaterials across the cell membranes into mitochondria, DNA damages, and the damage of cell membranes are the main causes of their toxicity, which ought to be comprehensively explored. In this study, recent advancements in diagnostic and therapeutic applications of functionalized Au and Ag nanomaterials are deliberated, focusing on important challenges and future directions.

## 1. Introduction

The functionalization of nanomaterials with suitable capping ligands or bioactive agents is an interesting strategy in designing nanosystems with suitable applicability and biocompatibility; the physicochemical and biological properties of these nanomaterials can be highly improved for biomedical applications [1,2,3,4]. In this context, numerous explorations have been performed in the functionalization of silver (Ag) and gold (Au) nanoparticles (NPs) with unique physicochemical properties to obtain high sensitivity and selectivity, biocompatibility, targeting features, and multifunctionality for biomedical purposes [5,6,7]. For instance, Au nanocontrast agents with high biocompatibility and optical properties have been applied for the sensitive detection of different diseases [8]. Additionally, folate-conjugated Au nanomaterials demonstrated excellent potential for targeted cancer therapy [9]. Nanorods (NRs) of Au could efficiently convert photon energy into heat, providing suitable hyperthermia effects to suppress tumor growth (in vitro and in vivo) [10]. Polycationic chitosan/Au NRs have also been designed with promising potential for the photothermal therapy and gene therapy of breast cancer [11].

Surface-functionalized Au NPs with distinctive optical, electrical, photothermal, and specific surface plasmon resonance features have been explored as strong colorimetric and fluorescent sensors that can provide immense opportunities for broad biomedical applications [12]. Different nanosystems have been constructed on the basis of Ag and Au NPs functionalized with a variety of compounds such as amino acids, polymers/biomolecules, fluorophores, and thiol-based organic molecules for cancer theranostics, sensing and imaging, and drug and gene delivery purposes. There are various chemical, physical, and bioecological methods for synthesizing these NPs with different sizes and morphologies, including nanolithography, thermolysis, milling, laser ablation, pyrolysis, chemical vapor deposition, electrochemical techniques, seeding-growth techniques, and microwave (MW)-assisted methods, among others [12,13,14]. Additionally, surface modifications of these NPs for specific functions have been performed using chemical reduction, secondary modification, physical sorption, green-based approaches, self-assembling of monolayers, polymer coating, covalent immobilization of ligands, bio-affinity immobilization of ligands, MW-assisted techniques, etc. The specific functionalization processes can help to improve the stability and biocompatibility of these nanomaterials in addition to reducing their aggregation and sedimentation [12,13,15,16,17]. Future studies should be undertaken toward the design of novel functionalization tactics to improve the properties of Au- and Ag-based nanosystems and reduce their toxicity (Table 1). The possible release of cytotoxic radicals and ions, the internalization of nanomaterials, the alteration of cellular signaling pathways, the translocation of these nanomaterials across the cell membranes into mitochondria, and the damage of cell membranes are the main causes of their toxicity, which should be comprehensively explored [18,19]. In this study, recent advancements in the biomedical applications of functionalized Au and Ag nanomaterials are deliberated, focusing on important challenges and future directions.

## 2. Therapeutics and Diagnostics

### 2.1. (Bio)imaging and (Bio)sensing

**Figure 1:**
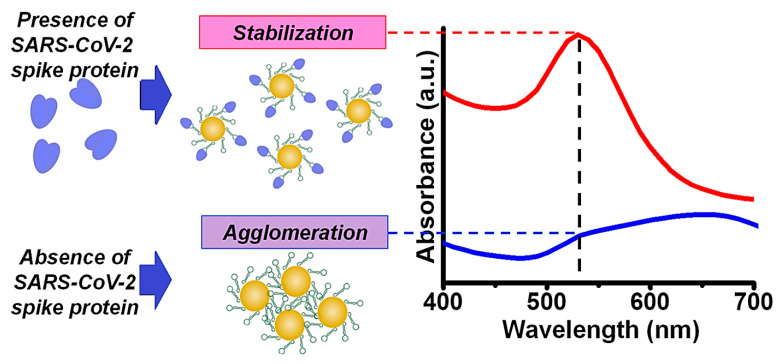
Specific detection of SARS-CoV-2 spike protein using aptamer-functionalized Au NPs. Adapted from Ref [41] with permission. Copyright 2021 Elsevier.

The visualization of Au nanomaterials can simply be performed by photometry and electron microscopy due to the high electron density, specific absorption, and scattering in the visible and near-infrared region [38]. These nanomaterials have been explored for (bio)imaging, (bio)sensing, and cellular labelling, providing excellent opportunities for the specific detection of various diseases (Table 2) [38,39]. The capping agents in biosensing can provide some benefits such as specific recognition, improved biocompatibility and biodegradability, electrostatic interactions, enhanced stability, and water solubility. For the detection of viral infections, Lew et al. [40] reported the construction of a novel nanosystem based on epitope-functionalized Au nanomaterials for the specific recognition of SARS-CoV-2 IgG antibodies, providing opportunities for detecting the SARS-CoV-2 infection with high specificity (100%) and sensitivity (83%) [40]. Similarly, an enzyme-linked immunosorbent assay was deployed for the detection of SARS-CoV-2 by applying aptamer-functionalized Au nanomaterials that specifically targeted the spike membrane protein of the virus (Figure 1) [41]. Consequently, the designed nanosystems detected 16 nM and higher concentrations of spike protein in phosphate-buffered saline using plasmon absorbance spectra; 3540 genome copies/μL of inactivated SARS-CoV-2 could be detected using this technique [41].

Citrate-functionalized Ag NPs were prepared for the sensitive detection of glutathione in human biological blood and serum samples using surface-enhanced infrared absorption spectroscopy [45]. Accordingly, silver NPs interacted with glutathione on the basis of structural orientation, energy, and affinity, offering a suitable method for the sensitive and selective determination of glutathione (the linear range = 10–100 μg mL^−1^ and correlation coefficient = 0.993); the limit of detection was ~1.74 μg mL^−1^ with a limit of quantification of ~5.30 μg mL^−1^ [45]. In another study, a surface-enhanced Raman spectroscopy (SERS)–based platform with high sensitivity was constructed utilizing SERS-active hollow polypyrrole nanohorn and peptide-functionalized Au NPs. Subsequently, silver was deposited on the inside and outside of the nanohorn surface to prepare this SERS-active platform, providing excellent opportunities for the specific detection of proteolytic biomarkers and for early diagnosis of diseases [47].

Biocompatible glycol chitosan–coated Au NPs were designed as photoacoustic contrast agents for the cellular imaging of cancers [48]. The prepared nanosystems exhibited suitable cellular uptake in breast cancer cells due to the synergistic effects of glycol chitosan and Au NPs, triggering enhanced photoacoustic signals in tissue-mimicking cell phantoms. These signals initiated from the plasmon coupling effect of these Au-based nanosystems after the cellular uptake in the cancerous cells [48]. Similarly, a nanosystem with high localization and an enhanced electromagnetic field was constructed from poly(perylene diimide), polyethylene glycol, and Au NPs. This nano-agent with improved optical features illustrated suitable applicability for photoacoustic imaging and photothermal therapy [49]. Additionally, a multifunctional nanohybrid system as a photosensitizer was constructed utilizing 4-mercaptobenzoic acid-capped Ag NPs with improved singlet oxygen formation and fluorescence features to be applied simultaneously for bioimaging and photodynamic ablation of HeLa cancer cells. As a result, this biocompatible nanosystem with enhanced photostability and negligible fluorescence quenching (~5%) could be deployed for cell tracking. The findings of this study demonstrated that this brightly fluorescent nano-agent had improved efficacy (~4 times higher than the control sample) in photodynamic therapy under low-intensity white light irradiation (40 mW cm^−2^) for ~10 min, offering a smart platform for advanced theranostics in future bio- and nanomedicine [50].

### 2.2. Drug and Gene Delivery

**Figure 2:**
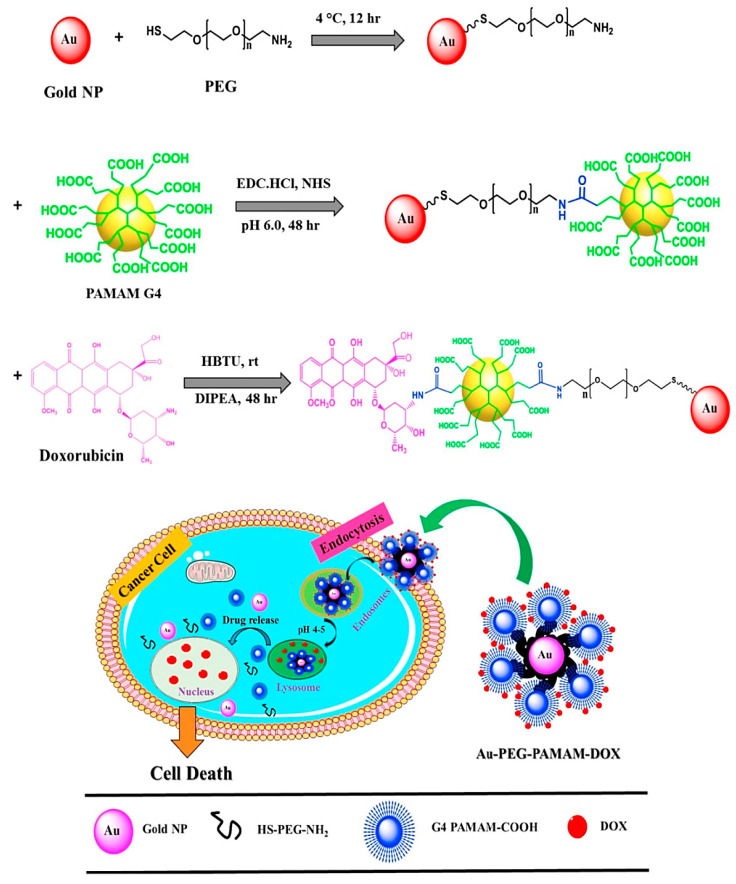
The preparative process and mechanisms of pH-triggered multifunctional nanosystems constructed from Au NPs, polyethylene glycol (PEG), and polyamidoamine (PAMAM) G4 dendrimer for targeted delivery of doxorubicin (DOX). 1-ethyl-3-(3-dimethylaminopropyl) carbodimide (EDC); O-(benzotriazol-1-yl)-*N*,*N*,*N′*,*N′*-tetramethyluronium hexafluorophosphate (HBTU); diisopropyl ethylamine (DIPEA). Adapted from Ref [15] with permission. Copyright 2017 Elsevier.

Au NPs with a large surface area can be deployed as effective platforms for therapeutic agents, allowing for the efficient binding of targeting agents and drugs. In one study, Au nanomaterials stabilized by pullulan were coupled with 5-fluorouracil and folic acid for targeted drug delivery and cancer imaging [28,51]. These nanosystems can be delivered to the cells via active or passive target delivery approaches. The passive targeting approach includes the deposition of the nanoparticles within the tumor’s irregular vasculature to permit the transfer of large particles through the endothelium. The passive targeting approach depends on the enhanced permeability and retention (EPR) effect; however, active targeting hinges on ligand–receptor binding precisely formed for targeting analytes to attain specificity and selectivity features. In one study, a drug delivery nanosystem was designed on the basis of multifunctional Au NPs for the intracellular delivery of a doxorubicin anticancer drug (Figure 2) [15]. The NPs were further stabilized with thiolated polyethylene glycol, and then covalently coupled with a polyamidoamine G4 dendrimer. Notably, it was indicated that polyethylene glycol polymer–coated Au NPs exhibited better colloidal stability and biocompatibility with negligible toxicity compared to citrate-coated Au NPs. Consequently, doxorubicin was efficiently and specifically delivered to the targeted cells, providing pH-triggered multifunctional nanoplatforms for the intracellular transferring of different anticancer agents [15].

RNA-conjugated Au NPs were fabricated to efficiently knock down the formation of luciferase in cancerous cell lines (HeLa cells) transfected luciferase. These conjugates exhibited a longer lifetime (~6 times longer) than the double-stranded RNA; in addition, the cells could simply penetrate with no need of deploying any transfection agents [52]. Moreover, Au NPs coated with a layer of DNA-capped quantum dots were loaded with doxorubicin for cancer therapy. Accordingly, the DNA sequence could bind to an mRNA-encoding MRP1 (a crucial factor in drug resistance) inside the cancerous cells to improve therapeutic efficacy [32]. Similarly, a biocompatible drug delivery nanosystem has been introduced for colorectal cancer therapy (in vivo) based on doxorubicin-loaded oligonucleotides attached to Au NPs (~13 nm) [53].

### 2.3. Cancer Diagnosis and Therapy

**Figure 3:**
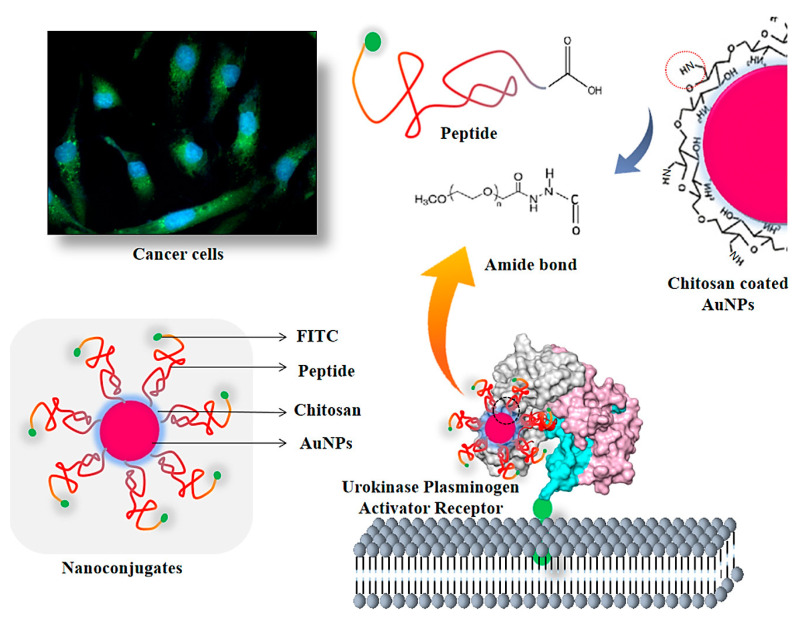
A nanosystem constructed from chitosan polymer and peptide-functionalized Au nanomaterials as a nanoprobe for specific detection and imaging of a urokinase plasminogen activator receptor in cancers. Adapted from Ref [59] with permission. Copyright 2021 Elsevier.

Currently, numerous in vitro and in vivo studies have been performed on cancer diagnosis and therapy applications of Au NPs due to their unique physicochemical properties and high versatility [54,55]. Functionalized Au NPs with high biocompatibility exhibited attractive potential in photothermal therapy and the thermal destruction of cancers [56]. For instance, Au NRs functionalized with folic acid were deployed for targeted delivery to melanoma cells [57]. The prepared nanosystem exhibited alluring possibilities for photothermal cancer therapy. As a result, apoptosis (~10.2%), necroptosis (~18.3%), and necrosis (~17.6%) of tumor cells could be obtained at lower temperatures, but after increasing the temperature to 49 °C, the pattern of cell death changed to necrosis-dominant (~52.8%) [57]. Moreover, fungal-crude-protein-extract–mediated Au nanomaterials (~19.72 nm) were functionalized with an aptamer to improve their cytotoxic effects on cancerous cells while reducing their toxicity in normal NIH3T3 cells. Consequently, the nanosystem exhibited improved accumulation in cancerous cells, thus providing nucleolin-targeted cell cytotoxicity via nucleus damage and stimulation of oxidative stress [58]. For the detection of cancer cells, a nanosystem was designed on the basis of fluorescently labeled peptide-functionalized Au nanomaterials and chitosan polymer for the specific targeting and imaging of urokinase plasminogen activator receptor positive cells (Figure 3). This nanosystem with improved uptake potential should be further explored for specific molecular targeting and imaging in cancers and tumors (especially in metastasis) [59].

A novel nanosystem was constructed via the conjugation of gadolinium (Gd)(III) complexes and prostate-specific-membrane-antigen–targeting ligands to the surfaces of Au NPs for targeted magnetic resonance–guided radiotherapy with improved targeting precision and efficacy. The designed nanocontrast agents exhibited robust magnetic resonance contrast and radiation therapy efficiency (in vitro and in vivo), providing encouraging nanosystems for cancer therapy and diagnosis with good tumor accumulation and high in vivo radiation dose amplification. However, toxicity and biocompatibility issues along with their clinical translation ought to be systematically addressed [60]. Similarly, Gd(III)-labeled DNA-Au NP conjugates were synthesized as multifunctional nanoprobes for cancer diagnostics (optical and magnetic resonance imaging probes). Compared to the molecular dithiolane-Gd(III) complexes, relaxivity was enhanced ~4.5-fold [61]. Additionally, prostate-specific membrane antigen (as a targeting ligand) was conjugated to Au NPs for localized prostate cancer therapy and diagnosis. Consequently, the designed nanosystems demonstrated enhanced radiotherapy efficacy with improved cellular uptake and tumor-targeting properties [62]. Yang et al. synthesized nanoprobes of Au/alpha-lactalbumin for the treatment and imaging of breast cancer, with a biodegradability and safety profile; this method showed great potential for the systemic recognition and targeted/localized treatment of different cancers [63].

### 2.4. Photothermal and Photodynamic Therapy

With their controlled treatment area of light exposure, photothermal (PTT) and photodynamic therapy (PDT) have been broadly applied in bio- and nanomedicine. The performance of PDT includes the excitation of a photosensitizer at a precise wavelength. The photosensitizer transfers its energy to the molecular oxygen and thus produces reactive oxygen species (ROS), destroying tumor cells through necrotic or apoptotic cell death pathways. PTT can be applied as a therapeutic method in which a therapeutic factor is stimulated with a specific wavelength of light and energy to create heat through photo absorption, thereby killing the surrounding cancer cells. In this method, a near-infrared laser (NIR) is applied to irradiate the tumor cells locally in order to uniformly enhance the temperature in the tumor and inhibit damage to healthy cells; effective ablation occurs when the center of the tumor reaches a therapeutic temperature above 55 °C [64,65].

Au NRs and nanoshells have been widely used in PTT because of their robust absorption in the NIR region [66]. Since Au NPs show a strong tendency to aggregate, researchers have overwhelmed this subject through functionalizing them with hydrophilic polymers, including chitosan, hyaluronic acid, and polyethylene glycol (PEG), to enhance stability and avoid decline of heat conversion properties. Moreover, these polymers increase the stability and solubility of NPs, nonetheless also making them suitable for PDT and PTT therapy. Au NPs were modified with chitosan and were connected to a PS porphyrin derivative, recognized as meso-tetrakis (4-sulphonatophenyl) porphyrin (TPPS), for PDT and PTT therapy. The NPs manufactured a good quantity of singlet oxygen and a high temperature of 55 °C, indicating that this drug has potential for use in tumor phototherapy applications [66].

Au NRs were modified with PEG toward the synthesis of PEG-Au NRs and conjugated by a TPS at the end of the PEG chain, thus creating TPS-PEG-Au NRs. Energy absorption under NIR-laser irradiation enhanced the temperature of the Au NRs and formed ROS derived from talaporfin sodium. The combination of nanoplatforms and NIR-laser irradiation showed outstanding cytotoxic effects against lung cancer cells under an NIR laser. Au NSs are effective multifunctional NPs simultaneously used in PTT and PDT using NIR-laser irradiation. The outcomes offer an advantageous model of nanomedicine for cancer therapy using NIR lasers [67]. Kayani et al. developed nanosystems constructed from PEG-curcumin (Cur)@Au NPs for PTT and sonodynamic therapy of melanoma cancer. The nanoplatform triggered localized hyperthermia and apoptosis of cancerous cells under PTT and ROS generated by sonodynamic therapy with synergistic effects [68].

The functionalization of nanomaterials with aptamers can offer a highly efficient diagnostic assay to probe target cells and tissues. Thus, various studies have recognized aptamers for imaging, detection, and intracellular analysis [69]. An aptamer-conjugated Au NRs/Ce6 complex was established to target cancer therapy. The Sgc8 aptamer can target leukemia T cells and is conjugated to Au NRs by covalent bonding. Short DNA sequences labelled with Ce6 hybridize with the aptamer on the surface of Au NRs, which causes fluorescence quenching due to the proximity of Ce6 to the gold surface. With the binding of aptamer and target cancer cells, Ce6 is released and functions as a PDT agent under NIR (812 nm) light irradiation. Also, in the study, the aptamer-Au NR conjugates significantly killed cancer cells by combining PDT and PTT under NIR irradiation, thus indicating their feasibility for multimodality targeted cancer therapy [70].

**Figure 4:**
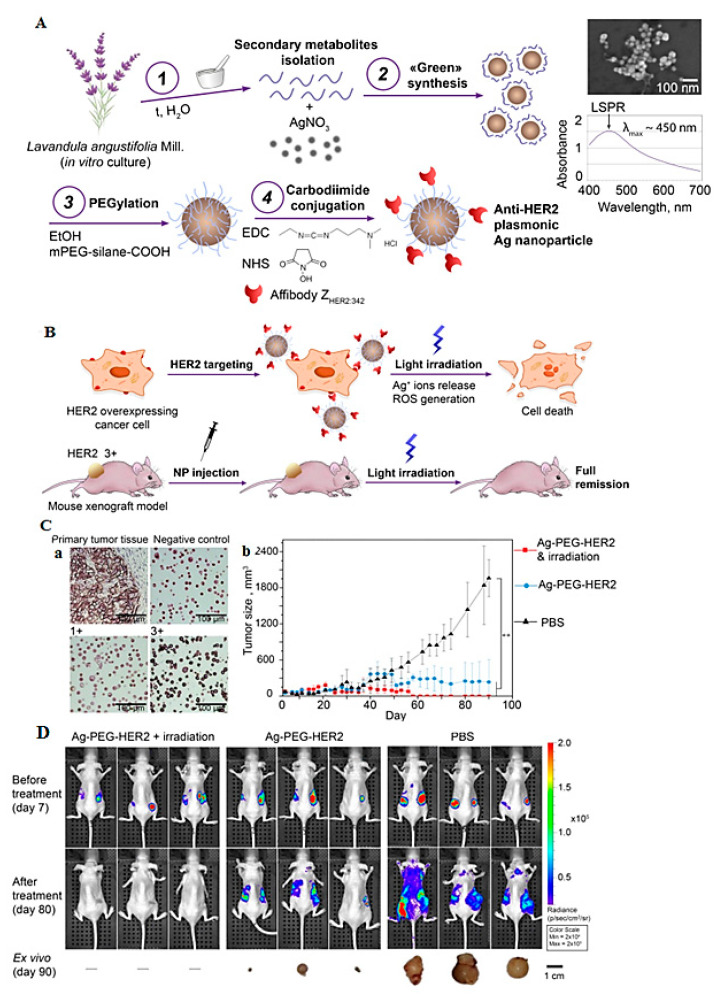
(**A**) The preparative process of Ag-PEG-HER2 NPs. (**B**) Photothermal-induced death of cancer cells via targeted delivery of functionalized Ag NPs to specific cells under the light irradiation, causing Ag^+^ ions release and ROS generation with total tumor elimination. (**C**-**a**) Immunohistochemistry of primary tumor tissue and Hercep Test controls: negative HER2 expression (1+) and HER2 overexpression (3+). (**C-b**) Dynamics of tumor growth under the treatment with injections of phosphate-buffered saline (PBS), Ag-PEG-HER2 NPs, and Ag-PEG-HER2 with 1 h post blue light irradiation. (**D**) Bioluminescent imaging of BT/NanoLuc xenograft tumors before treatment on day 7 and after treatment on day 80, as well as ex vivo imaging of primary tumors (day 90). Adapted from Ref [71] with permission (CC BY) Copyright 2022 Multidisciplinary Digital Publishing Institute (MDPI).

Although Au-based NPs are the most widely examined materials for PTT, recent studies have revealed that this approach may also be suitable for the application of Ag NPs, as they have the strongest light-scattering and surface plasmon [71]. Researchers have developed chitosan-modified Ag nano-triangles (NTs) that exhibited high specificity to the NCI-H460 cell line. An 80% decrease in cell viability was observed with 0.3 μg mL^−1^ Ag NTs and an 800 nm laser, although the viability of the cells pre-incubated with gold nanorods and subjected to NIR stimulation was only reduced by 20% [72]. Ag NPs with triangular shapes could be applied for PTT against MCF7 and MDA-MB-231 breast cancer cells, with a concentration of ~50 μg mL^−1^ under irradiation at 800 nm laser [73]. In addition, Ag-PEG-HER2 NPs were designed for the PTT of cancers. The 35-nm-size modified NPs were synthesized through grinding with *Lavandula angustifolia Mill* extracts (Figure 4A), which exhibited a hypothermic effect under light radiation treatment. Light irradiation effectively generated ROS with a cytotoxic effect of the HER2-targeted Ag NPs. Tumor growth inhibition upon treatment with the HER2-targeted Ag NPs could be obtained, inhibiting the metastatic spread after the treatment. These NPs have enormous potential for the photothermal therapeutic treatment of HER2-overexpressing cancers [71].

### 2.5. Tissue Engineering and Regenerative Medicine

Endolichenic fungus–derived anti-quorum–sensing chrysophanol was decorated on Ag nanomaterials to produce anti-adhesion and anti-biofouling coating materials against bacterial invasion, offering enhanced and long-term prevention of bacterial adhesion and subsequent colonization in urinary catheters [74]. Accordingly, these biocompatible nanosystems could efficiently affect the lipopolysaccharide formation, hydrophobicity of surface, and virulence gene expression of bacterial biofilm cells, as well as environmental DNA content, thus reducing the invasion and generation of biofilms (in vivo) [74]. Similarly, *Terminalia catappa*–functionalized silver NPs were prepared against multidrug-resistant *Pseudomonas aeruginosa*, *Candida albicans*, and methicillin-resistant *Staphylococcus aureus* [75]. This functionalized nanosystem exhibited high inhibitory effects against biofilm generation, showing inhibitory effects against the colonization and adherence of biofilm-forming cells. The possible mechanisms included structural changes (such as disintegration, separation, and deformation) in bacterial cell walls and membranes, which caused cell death by considerable loss of membranes or the integrity of cell walls [75].

In addition, PEG-Ag NPs were synthesized with enhanced bactericidal effects toward *S. aureus*, in which an increase in hydroxyl ions on the surface of the silver NPs could be the reason for their improved bactericidal effect [76]. Impressively, Au NPs were conjugated with the vascular endothelial growth factor-A_165_ (a proangiogenic growth factor) and (11-mercaptoundecyl)-*N*,*N*,*N*-trimethylammonium cation (with antibacterial activity) to produce nanosystems with dual functions including antimicrobial and proangiogenic performances for wound healing in diabetic mice [77]. Consequently, these nano-agents exhibited high antimicrobial activities against multidrug-resistant bacteria such as methicillin-resistant *S. aureus*. They showed robust bactericidal activities (in vivo) and enhanced the generation of collagen fibers and epithelialization, offering favorable materials for wound healing and treating chronic wound infections [77].

**Figure 5:**
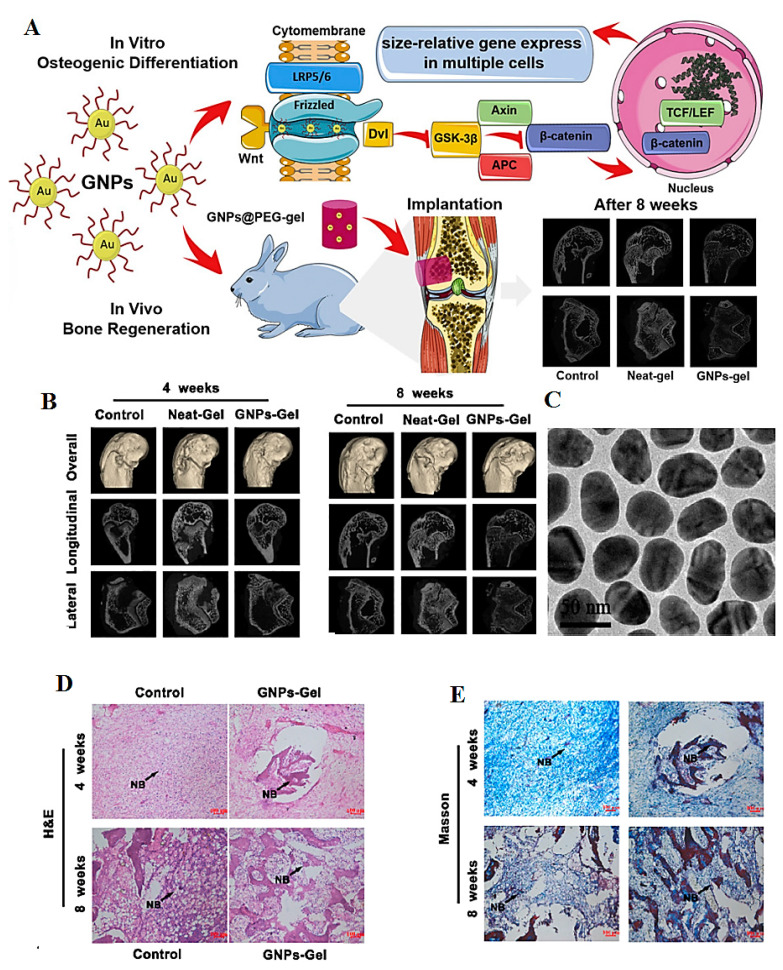
(**A**) Schematic illustration of the role of PEGylated Au NPs in improving osteogenic differentiation. (**B**) Three-dimensional (3D) micro-computed tomography (CT) images of femoral condyle at 4 and 8 weeks after being treated with neat-gel and Au NPs-gel, respectively. (**C**) Transmission electron microscopy (TEM) images of PEGylated Au NPs with a size of 45 nm. (**D**,**E**) Staining images of a bone defect implanted with PEG-gel and Au NPs-45 nm@PEG-gel on weeks 4 and 8, respectively. Au NPs: GNPs. Adapted from Ref [82] with permission. Copyright 2020 Elsevier.

Au NPs have been established as promising nanomaterials for bone regeneration. These NPs can improve the osteogenic differentiation of mesenchymal stem cells, act as osteogenic agents, increase the grafting of bone implants, and hasten bone formation in bone defects [78,79]. Results have illustrated that hydrogels loaded with Au NPs can increase the differentiation, proliferation, and alkaline phosphate activities of human adipose–derived stem cells to be differentiated into osteoblast cells [80]. Chitosan-conjugated Au NPs affected bone differentiation. Positively charged NPs with a hydrodynamic diameter of 40 nm were formed through the chitosan reduction assay. The results revealed that Au NPs conjugated with chitosan can enhance the expression of marker genes related to bone differentiation in mesenchymal stem cells derived from human fat at low concentrations. They illustrated that such nanostructures can improve osteogenesis through the Wnt/β-catenin signaling pathway and can be deployed in bone tissue engineering [81]. PEGylated Au NPs with a size of 45 nm showed good biocompatibility and osteogenic differentiation on MC3T3-E1 cells. PEGylated Au NPs can increase alkaline phosphatase (ALP) activity, the expression of osteogenic marker genes, and up-regulated β-catenin and p-GSK-3β genes (Figure 5). In addition, Au NPs @PEG-gel encouraged bone regeneration effectively in vivo. This evaluation of the effects of functionalized Au NPs on osteogenic differentiation and bone regeneration could shed light on their clinical translational applications [82].

Epigallocatechin gallate (EGCG) is the most prevalent polyphenol in green tea and can effectually scavenge ROS and can reduce autoimmune arthritis by moderating Th17/Treg. EGCG can inhibit osteoporosis in an in vivo model and display a therapeutic effect on periodontitis in rats [83]. EGCG-Au NPs can be applied as an anti-osteoclastogenic drug. These nanoplatforms can be effectively taken into the cell and placed in the intracellular compartment, where they are overwhelmed by endocytosis. Free EGCG can prevent the osteoclast differentiation of bone marrow–derived macrophages by suppressing ROS and a related mitogen activated protein kinase (MAPK)–signaling pathway. EGCG-Au NPs also display excellent antioxidant activity in vitro. In vivo studies exhibited that EGCG-Au NPs can improve bone mineral density and prevent bone resorption. Accordingly, bioactive EGCG-Au NPs can be applied as therapeutic agents in the treatment of inflammatory osteolysis and other bone diseases related to bone loss; these NPs (~30 nm) displayed highly efficient anti-osteoclast effects compared to free EGCG l [84].

## 3. Challenges and Opportunities

Despite the various advantages of surface-functionalized Au- and Ag-based nanomaterials, the use of these NPs systems for tissue targeting in the human body or in vivo has not been more generally studied relative to other nanomaterials, such as lipid-based systems [85]. Considering the biomedical applications of Au- and Ag-based nanocarriers as drug delivery systems as well as their in vivo studies, it is very important to evaluate their behavior in biological body fluids. Since most of the metal NP–based systems are formulated for intravenous administration, their first contact is blood plasma with biological substances [86]. These systems may cause adverse responses, namely the accumulation of nanocarriers and rapid blood flow clearance, as well as inflammatory responses in the body. In addition, several important challenges regarding the targeting properties and related nano–bio interactions after the incorporation of NPs in blood circulation ought to be considered [87]. In this context, designing nanomaterials with optimal physicochemical properties, including size, morphology, and charge, along with surface functionalization, can significantly help to solve these challenges. For instance, after evaluating the differences in the interaction of Au and Ag NPs with glycosylated vs. non-glycosylated transferrin, it was revealed that the binding strength between nanomaterials and transferrin and the alterations in the secondary protein structure largely depend not only on the physicochemical features of nanomaterials but also on their protein glycosylation status [88,89]. Notably, functionalized NPs should first be evaluated in an animal model using a plasma cell line type to ensure the safe transferability of these unique drug delivery systems from animal experiments to human in vivo experiments. Furthermore, similar animal experiments, at least in vitro experiments and blood plasma studies, should be performed with human cell lines, and the obtained results should be compared in relevant animal studies. This can provide possible predictions about whether the stability of metal NPs in the blood is the same in human systems. Ideally, this is the most appropriate way to smoothly transfer surface-functionalized NPs from animal models to human clinical trials [85].

Another challenge is finding the mechanisms and specific roles of functionalized Au and Ag NPs in tissue engineering [75]. These are predominantly helpful since they can deliver bioactive molecules along with diagnosing stem cell differentiation while increasing stem cell differentiation and cell–cell interactions. However, some important challenging questions regarding the presence of unreliable results related to the interaction between cells and Au NPs are still unanswered [90]. While it has been stated that Au NPs can contribute to more ROS production, it has recently been reported that ROS production is reduced when Au NPs are incorporated into scaffolds. Notably, studies have illustrated that Au NPs could stimulate osteogenesis of stem cells while inhibiting adipogenic differentiation. Thus, finding the possible reactions of cells with Au NPs is necessary. Future courses ought to combine both in vivo and in vitro assays in a long-term study to culminate in standardized procedures such as appropriate cell type, improved Au NP dose, and cytotoxicity assays [91]. In one study, Au NPs were functionalized with polyethyleneimine and polyethylene glycol to evaluate their neuronal toxicity and the cellular/sub-organ biodistribution. Accordingly, the surface functionalization could significantly enhance the biocompatibility and biodistribution of these NPs; size and surface chemistry were crucial factors affecting the nanotheranostic potential of the NPs [92].

## 4. Conclusions and Future Outlook

Functionalized nanomaterials have been widely explored in the fields of drug and gene delivery, cancer theranostics, imaging and diagnosis, and anti-infections. Among the nanomaterials, functionalized Au/Ag-based nanosystems possess the special benefits of improved biocompatibility, good biodegradability, targeting, specificity and selectivity, low toxicity, high drug loading capacity, and sustained and controlled drug release behavior. Surface functionalization of these nano-agents can help to overcome concerns about biosafety, thus providing excellent opportunities for developing smart nanosystems with gene and drug delivery, bioimaging and biosensing, and cancer theranostics applicability. However, more elaborative studies should still be conducted to fill the existing research gaps, especially pertaining to nanotoxicological assessments, optimization processes, and related mechanisms.

The selection of suitable capping agents with multifunctional features, as well as surface modification techniques, can play important roles in the development of these Ag- and Au-based nanosystems. Notably, the majority of studies in this field are focused on the laboratory scale; thus, future explorations should be moved toward the large-scale production and functionalization of these nanomaterials utilizing appropriate capping agents. Toxicity and biosafety assessments are vital in acquiring multi-functionalized nanosystems with high efficiency and low or negligible adverse side effects and should be comprehensively performed; this is particularly true for clinical translational studies.

## Figures and Tables

**Table 1:** Functionalized Ag and Au NPs with versatile diagnostic and therapeutic applications.

NPs	Functional or Capping Agents	Drugs/Therapeutic Agents	Applications	Refs.
**Ag**	Polyvinyl alcohol and chitosan	Naproxen	Strong inhibitory effects against biofilm generation and sustained drug release	[20]
	Polyvinyl alcohol	Doxorubicin and curcumin	Anticancer and antibacterial applications; drug delivery system	[21]
	Polyethylene glycol	I-131 radionuclide	Tumor theranostic guided drug delivery	[22]
	Polyvinyl alcohol, polyvinylpyrrolidone, pectin	Mafenide acetate	Skin wound healing and antibacterial activity	[23]
	Chitosan	Ag/chitosan	Burn wound healing	[24]
	Bioactive agents from *Aesculus hippocastanum*	Resveratrol, bioactive agents from plant leaf extract	Antibacterial, antioxidant, and drug release system activities	[25]
	Nucleotide (adenosine triphosphate)	-	Antimicrobial and anticancer effects	[26]
**Au**	Polyethylene glycol and polyamidoamine G4 dendrimer	Doxorubicin	pH-triggered intracellular anticancer drug release	[15]
	Bovine serum albumin	Methotrexate	Anticancer drug delivery system	[27]
	Pullulan	5-fluorouracil and folic acid	Anticancer drug delivery system	[28]
	Heavy metal binding proteins in recombinant *Escherichia coli*	Doxorubicin	Anticancer effects; drug delivery system	[29]
	_L_-cysteine methyl ester hydrochloride conjugated to poly(ethylene glycol)	Small interfering RNA (siRNA)	Gene delivery (in vitro and in vivo) and cancer therapy	[30]
	Capped with glutathione and conjugated with a CALNN peptide	Linalool	Cancer therapy (ovarian cancer cells, SKOV-3)	[31]
	A layer of DNA-capped quantum dots	Doxorubicin; DNA sequence	Cancer therapy; doxorubicin-resistant cell line	[32]
	_L_-Cysteine methyl ester hydrochloride	Targeted delivery of plasmid DNA encoded p53 gene	Cancer therapy and gene delivery	[33]
	Poly di(carboxylatophenoxy)phosphazene nanospheres	-	Computed tomography and photoacoustic imaging	[34]
	Folate	-	Targeted bioimaging	[7]
	Polycaffeic acid/folate	Bortezomib	Cancer therapy; tumor inhibition	[35]
**Au and Ag**	Bioactive agents from *Butea monosperma* leaf extract	Doxorubicin	Cancer therapeutics	[36]
**Bimetallic Au-Ag**	Folic acid, poly(amidoamine) dendrimers	-	Targeted computed tomography imaging of cancer cells (in vitro)	[37]

**Table 2:** Some selected examples of functionalized Ag and Au NPs with (bio)sensing applications.

NPs	Functional Agents	Applications	Remarks	Refs.
**Au**	Aptamer	Colorimetry; detection of *Escherichia coli* O157:H7	Au NP-based colorimetric biosensing with flexibility for specific diagnosis; high sensitivity	[42]
	Aptamer	Surface-enhanced Raman scattering (SERS) biosensor; label-free detection of interleukin-6 (IL-6) in serum	Quantitative analysis of IL-6 in 10^−12^–10^−7^ M range can be obtained; the aptamer-SERS assay detected IL-6 in blood with lower limit of detection of 1 pM	[43]
	Epitope	Detection of SARS-CoV-2 IgG antibodies	Excellent specificity (~100%) and sensitivity (~83%)	[40]
	Aptamer	Detection of SARS-CoV-2 spike proteins	Specific detection of pathogenic virus; 16 nM and higher concentrations of spike protein could be detected in phosphate-buffered saline using plasmon absorbance spectra; 3540 genome copies/μL of inactivated SARS-CoV-2 could be recognized	[41]
	Peptide aptamers	Biosensing of cancer biomarker (Mdm2), in vitro	Excellent chemical and colloidal stability; high sensitivity	[44]
**Ag**	Citrate	Specific detection of glutathione	Selective detection of glutathione; the linear range was 10–100 μg mL^−1^ and the correlation coefficient was 0.993; the limit of detection was ~1.74 μg mL^−1^; the limit of quantification was ~5.30 μg mL^−1^	[45]
	Aptamer	For specific detection of Michigan cancer foundation-7 (MCF-7) human breast cancer cells and MUC1 biomarker	The sensor could detect MCF-7 cells in the concentration range from 1.0 × 10^2^ to 1.0 × 10^7^ cells mL^−1^ with a detection limit of 25 cells	[46]

## Data Availability

Not applicable.

## References

[1] Iravani S., Abd-Elsalam K.A. (2020). Core-shell hybrid nanoparticles: Production and application in agriculture and the environment. Multifunctional Hybrid Nanomaterials for Sustainable Agri-Food and Ecosystems: Micro and Nano Technologies.

[2] Iravani S., Jamalipour Soufi G., Shukla A.K. (2019). Gold Nanostructures in Medicine and Biology. Nanoparticles in Medicine.

[3] Nasrollahzadeh M., Sajjadi M., Iravani S., Varma R.S. (2020). Trimetallic Nanoparticles: Greener Synthesis and Their Applications. Nanomaterials.

[4] Nasrollahzadeh M., Sajjadi M., Iravani S., Varma R.S. (2021). Green-synthesized nanocatalysts and nanomaterials for water treatment: Current challenges and future perspectives. J. Hazard. Mater..

[5] Huang X., Jain P., El-Sayed I., El-Sayed M. (2007). Special Focus: Nanoparticles for Cancer Diagnosis & Therapeutics-Review; Gold nanoparticles: Interesting optical properties and recent applications in cancer diagnostics and therapy. Nanomedicine.

[6] Luo D., Wang X., Zeng S., Ramamurthy G., Burda C., Basilion J.P. (2019). Targeted gold nanocluster-enhanced radiotherapy of prostate cancer. Small.

[7] Pyo K., Ly N.H., Yoon S.Y., Shen Y., Choi S.Y., Lee S.Y., Joo S.W., Lee D. (2017). Highly luminescent folate-functionalized Au22 nanoclusters for bioimaging. Adv. Healthc. Mater..

[8] Dykman L., Khlebtsov N. (2012). Gold nanoparticles in biomedical applications: Recent advances and perspectives. Chem. Soc. Rev..

[9] Samadian H., Hosseini-Nami S., Kamrava S.K., Ghaznavi H., Shakeri-Zadeh A. (2016). Folate-conjugated gold nanoparticle as a new nanoplatform for targeted cancer therapy. J. Cancer Res. Clin. Oncol..

[10] Turcheniuk K., Dumych T., Bilyy R., Turcheniuk V., Bouckaert J., Vovk V., Chopyak V., Zaitsev V., Mariot P., Prevarskaya N. (2016). Plasmonic photothermal cancer therapy with gold nanorods/reduced graphene oxide core/shell nanocomposites. RSC Adv..

[11] Dai X., Zhao X., Liu Y., Chen B., Ding X., Zhao N., Xu F.-J. (2021). Controlled Synthesis and Surface Engineering of Janus Chitosan-Gold Nanoparticles for Photoacoustic Imaging-Guided Synergistic Gene/Photothermal Therapy. Small.

[12] Chatterjee S., Lou X.-Y., Liang F., Yang Y.-W. (2022). Surface-functionalized gold and silver nanoparticles for colorimetric and fluorescent sensing of metal ions and biomolecules. Coord. Chem. Rev..

[13] Ielo I., Rando G., Giacobello F., Sfameni S., Castellano A., Galletta M., Drommi D., Rosace G., Plutino M.R. (2021). Synthesis, Chemical–Physical Characterization, and Biomedical Applications of Functional Gold Nanoparticles: A Review. Molecules.

[14] Ojea-Jiménez I., Capomaccio R., Osório I., Mehn D., Ceccone G., Hussain R., Siligardi G., Colpo P., Rossi F., Gilliland D. (2018). Rational design of multi-functional gold nanoparticles with controlled biomolecule adsorption: A multi-method approach for in-depth characterization. Nanoscale.

[15] Khutale G.V., Casey A. (2017). Synthesis and characterization of a multifunctional gold-doxorubicin nanoparticle system for pH triggered intracellular anticancer drug release. Eur. J. Pharm. Biopharm..

[16] Iravani S., Soufi G.J. (2021). Algae-derived materials for tissue engineering and regenerative medicine applications: Current trends and future perspectives. Emergent Mater..

[17] Iravani S., Varma R.S. (2019). Plant-derived Edible Nanoparticles and miRNAs: Emerging Frontier for Therapeutics and Targeted Drug-delivery. ACS Sustain. Chem. Eng..

[18] Luo D., Wang X., Burda C., Basilion J.P. (2021). Recent Development of Gold Nanoparticles as Contrast Agents for Cancer Diagnosis. Cancers.

[19] Tabish T.A., Dey P., Mosca S., Salimi M., Palombo F., Matousek P., Stone N. (2020). Smart gold nanostructures for light mediated cancer theranostics: Combining optical diagnostics with photothermal therapy. Adv. Sci..

[20] Mishra S., Teotia A.K., Kumar A., Kannan S. (2017). Mechanically tuned nanocomposite coating on titanium metal with integrated properties of biofilm inhibition, cell proliferation, and sustained drug delivery. Nanomedicine.

[21] Nguyen N., Le C.H. (2021). Synthesis of PVA encapsulated silver nanoparticles as a drug delivery system for doxorubicin and curcumin. Int. J. High Sch. Res..

[22] Sakr T.M., Khowessah O.M., Motaleb M.A., Abd El-Bary A., El-Kolaly M.T., Swidan M.M. (2018). I-131 doping of silver nanoparticles platform for tumor theranosis guided drug delivery. Eur. J. Pharm. Sci..

[23] Alipour R., Khorshidi A., Shojaei A.F., Mashayekhi F., Moghaddam M.J.M. (2019). Skin wound healing acceleration by Ag nanoparticles embedded in PVA/PVP/Pectin/Mafenide acetate composite nanofibers. Polym. Test..

[24] Oryan A., Alemzadeh E., Tashkhourian J., Ana S.F.N. (2018). Topical delivery of chitosan-capped silver nanoparticles speeds up healing in burn wounds: A preclinical study. Carbohydr. Polym..

[25] Kup F.O., Coskuncay S., Duman F. (2020). Biosynthesis of silver nanoparticles using leaf extract of Aesculus hippocastanum (horse chestnut): Evaluation of their antibacterial, antioxidant and drug release system activities. Mater. Sci. Eng. C.

[26] Datta L.P., Chatterjee A., Acharya K., De P., Das M. (2017). Enzyme responsive nucleotide functionalized silver nanoparticles with effective antimicrobial and anticancer activity. New J. Chem..

[27] Murawala P., Tirmale A., Shiras A., Prasad B.L.V. (2014). In situ synthesized BSA capped gold nanoparticles: Effective carrier of anticancer drug methotrexate to MCF-7 breast cancer cells. Mater. Sci. Eng. C.

[28] Ganeshkumar M., Ponrasu T., Raja D.M., Subamekala M.K., Suguna L. (2014). Green synthesis of pullulan stabilized gold nanoparticles for cancer targeted drug delivery. Spectrochim. Acta Part A: Mol. Biomol. Spectrosc..

[29] Seo J.M., Kim E.B., Hyun M.S., Kim B.B., Park T.J. (2015). Self-assembly of biogenic gold nanoparticles and their use to enhance drug delivery into cells. Colloids Surf. B Biointerfaces.

[30] Rahme K., Guo J., Holmes J.D., O’Driscoll C.M. (2015). Evaluation of the physicochemical properties and the biocompatibility of polyethylene glycol-conjugated gold nanoparticles: A formulation strategy for siRNA delivery. Colloids Surf. B Biointerfaces.

[31] Jabir M., Sahib U.I., Taqi Z., Taha A., Sulaiman G., Albukhaty S., Al-Shammari A., Alwahibi M., Soliman D., Dewir Y.H. (2020). Linalool-loaded glutathione-modified gold nanoparticles conjugated with CALNN peptide as apoptosis inducer and NF-κB translocation inhibitor in SKOV-3 cell line. Int. J. Nanomed..

[32] Zhang L., Jean S.R., Li X., Sack T., Wang Z., Ahmed S., Chan G., Das J., Zaragoza A., Sargent E.H. (2018). Programmable Metal/Semiconductor Nanostructures for mRNA-Modulated Molecular Delivery. Nano Lett..

[33] Abdel-Rashid R.S., Omar S.M., Teiama M.S., Khairy A., Magdy M., Anis B. (2019). Fabrication of gold nanoparticles in absence of surfactant as in vitro carrier of plasmid DNA. Int. J. Nanomed..

[34] Cheheltani R., Ezzibdeh R.M., Chhour P., Pulaparthi K., Kim J., Jurcova M., Hsu J.C., Blundell C., Litt H.I., Ferrari V.A. (2016). Tunable, biodegradable gold nanoparticles as contrast agents for computed tomography and photoacoustic imaging. Biomaterials.

[35] Aguilar L.E., Chalony C., Kumar D., Park C.H., Kim C.S. (2021). Phenol-Boronic surface functionalization of gold nanoparticles; to induce ROS damage while inhibiting the survival mechanisms of cancer cells. Int. J. Pharm..

[36] Patra S., Mukherjee S., Barui A.K., Ganguly A., Sreedhar B., Patra C.R. (2015). Green synthesis, characterization of gold and silver nanoparticles and their potential application for cancer therapeutics. Mater. Sci. Eng. C.

[37] Liu H., Shen M., Zhao J., Zhu J., Xiao T., Cao X., Zhang G., Shi X. (2013). Facile formation of folic acid-modified dendrimer-stabilized gold-silver alloy nanoparticles for potential cellular computed tomography imaging applications. Analyst.

[38] Liu L., Jiang H., Wang X. (2021). Functionalized gold nanomaterials as biomimetic nanozymes and biosensing actuators. TrAC Trends Anal. Chem..

[39] Murali K., Neelakandan M.S., Thomas S. (2018). Biomedical applications of gold nanoparticles. JSM Nanotechnol. Nanomed..

[40] Lew T.T.S., Aung K.M.M., Ow S.Y., Amrun S.N., Sutarlie L., Ng L.F.P., Su X. (2021). Epitope-Functionalized Gold Nanoparticles for Rapid and Selective Detection of SARS-CoV-2 IgG Antibodies. ACS Nano.

[41] Aithal S., Mishriki S., Gupta R., Sahu R.P., Botos G., Tanvir S., Hanson R.W., Puri I.K. (2022). SARS-CoV-2 detection with aptamer-functionalized gold nanoparticles. Talanta.

[42] Xie Y., Huang Y., Li J., Wu J. (2021). A trigger-based aggregation of aptamer-functionalized gold nanoparticles for colorimetry: An example on detection of Escherichia coli O157:H7. Sens. Actuators B Chem..

[43] Muhammad M., Shao C.-S., Huang Q. (2021). Aptamer-functionalized Au nanoparticles array as the effective SERS biosensor for label-free detection of interleukin-6 in serum. Sens. Actuators B Chem..

[44] Retout M., Blond P., Jabin I., Bruylants G. (2021). Ultrastable PEGylated Calixarene-Coated Gold Nanoparticles with a Tunable Bioconjugation Density for Biosensing Applications. Bioconjug. Chem..

[45] Khalkho B.R., Kurrey R., Deb M.K., Karbhal I., Sahu B., Sinha S., Sahu Y.K., Jain V.K. (2021). A simple and convenient dry-state SEIRS method for glutathione detection based on citrate functionalized silver nanoparticles in human biological fluids. New J. Chem..

[46] Yazdanparast S., Benvidi A., Banaei M., Nikukar H., Tezerjani M.D., Azimzadeh M. (2018). Dual-aptamer based electrochemical sandwich biosensor for MCF-7 human breast cancer cells using silver nanoparticle labels and a poly(glutamic acid)/MWNT nanocomposite. Microchim. Acta.

[47] Choi J.H., El-Said W.A., Choi J.-W. (2020). Highly sensitive surface-enhanced Raman spectroscopy (SERS) platform using core/double shell (Ag/polymer/Ag) nanohorn for proteolytic biosensor. Appl. Surf. Sci..

[48] Sun I.C., Ahn C.H., Kim K., Emelianov S. (2019). Photoacoustic imaging of cancer cells with glycol-chitosan-coated gold nanoparticles as contrast agents. J. Biomed. Opt..

[49] Yang Z., Song J., Dai Y., Chen J., Wang F., Lin L., Liu Y., Zhang F., Yu G., Zhou Z. (2017). Self-assembly of semiconducting-plasmonic gold nanoparticles with enhanced optical property for photoacoustic imaging and photothermal therapy. Theranostics.

[50] Yaraki M.T., Pan Y., Hu F., Yu Y., Liu B., Tan Y.N. (2020). Nanosilver-enhanced AIE photosensitizer for simultaneous bioimaging and photodynamic therapy. Mater. Chem. Front..

[51] Ganie S.A., Rather L.J., Li Q. (2021). A review on anticancer applications of pullulan and pullulan derivative nanoparticles. Carbohydr. Polym. Technol. Appl..

[52] Giljohann D.A., Seferos D.S., Prigodich A.E., Patel P.C., Mirkin C.A. (2009). Gene regulation with polyvalent siRNA− nanoparticle conjugates. J. Am. Chem. Soc..

[53] Lee C.-S., Kim H., Yu J., Yu S.H., Ban S., Oh S., Jeong D., Im J., Baek M.J., Kim T.H. (2017). Doxorubicin-loaded oligonucleotide conjugated gold nanoparticles: A promising in vivo drug delivery system for colorectal cancer therapy. Eur. J. Med. Chem..

[54] Medici S., Peana M., Coradduzza D., Zoroddu M.A. (2021). Gold nanoparticles and cancer: Detection, diagnosis and therapy. Semin. Cancer Biol..

[55] Xie S., Ai L., Cui C., Fu T., Cheng X., Qu F., Tan W. (2021). Functional Aptamer-Embedded Nanomaterials for Diagnostics and Therapeutics. ACS Appl. Mater. Interfaces.

[56] Abadeer N.S., Murphy C.J. (2016). Recent Progress in Cancer Thermal Therapy Using Gold Nanoparticles. J. Phys. Chem. C.

[57] Zhang Y., Zhan X., Xiong J., Peng S., Huang W., Joshi R., Cai Y., Liu Y., Li R., Yuan K. (2018). Temperature-dependent cell death patterns induced by functionalized gold nanoparticle photothermal therapy in melanoma cells. Sci. Rep..

[58] Saravanakumar K., Sathiyaseelan A., Mariadoss A.V.A., Hu X., Venkatachalam K., Wang M.-H. (2021). Nucleolin targeted delivery of aptamer tagged Trichoderma derived crude protein coated gold nanoparticles for improved cytotoxicity in cancer cells. Process Biochem..

[59] Shahdeo D., Kesarwani V., Suhag D., Ahmed J., Alshehri S.M., Gandhi S. (2021). Self-assembled chitosan polymer intercalating peptide functionalized gold nanoparticles as nanoprobe for efficient imaging of urokinase plasminogen activator receptor in cancer diagnostics. Carbohydr. Polym..

[60] Luo D., Johnson A., Wang X., Li H., Erokwu B.O., Springer S., Lou J., Ramamurthy G., Flask C.A., Burda C. (2020). Targeted Radiosensitizers for MR-Guided Radiation Therapy of Prostate Cancer. Nano Lett..

[61] Rotz M.W., Holbrook R.J., MacRenaris K.W., Meade T.J. (2018). A markedly improved synthetic approach for the preparation of multifunctional Au-DNA nanoparticle conjugates modified with optical and mr imaging probes. Bioconjug. Chem..

[62] Luo D., Wang X., Zeng S., Ramamurthy G., Burda C., Basilion J.P. (2019). Prostate-specific membrane antigen targeted gold nanoparticles for prostate cancer radiotherapy: Does size matter for targeted particles?. Chem. Sci..

[63] Yang J., Wang T., Zhao L., Rajasekhar V.K., Joshi S., Andreou C., Pal S., Hsu H.-t., Zhang H., Cohen I.J. (2020). Gold/alpha-lactalbumin nanoprobes for the imaging and treatment of breast cancer. Nat. Biomed. Eng..

[64] Abrahamse H., Hamblin M.R. (2016). New photosensitizers for photodynamic therapy. Biochem. J..

[65] Correia J.H., Rodrigues J.A., Pimenta S., Dong T., Yang Z. (2021). Photodynamic Therapy Review: Principles, Photosensitizers, Applications, and Future Directions. Pharmaceutics.

[66] Hirsch L.R., Stafford R.J., Bankson J.A., Sershen S.R., Rivera B., Price R.E., Hazle J.D., Halas N.J., West J.L. (2003). Nanoshell-mediated near-infrared thermal therapy of tumors under magnetic resonance guidance. Proc. Natl. Acad. Sci. USA.

[67] Kondo Y., Tagami T., Ozeki T. (2021). Fabrication of photosensitizer-polyethylene glycol-conjugated gold nanostars for simultaneous photothermal and photodynamic cancer therapy under near-infrared laser irradiation. J. Drug Deliv. Sci. Technol..

[68] Kayani Z., Vais R.D., Soratijahromi E., Mohammadi S., Sattarahmady N. (2021). Curcumin-gold-polyethylene glycol nanoparticles as a nanosensitizer for photothermal and sonodynamic therapies: In vitro and animal model studies. Photodiagn. Photodyn. Ther..

[69] Mahmoudpour M., Ding S., Lyu Z., Ebrahimi G., Du D., Dolatabadi J.E.N., Torbati M., Lin Y. (2021). Aptamer functionalized nanomaterials for biomedical applications: Recent advances and new horizons. Nano Today.

[70] Wang J., You M., Zhu G., Shukoor M.I., Chen Z., Zhao Z., Altman M.B., Yuan Q., Zhu Z., Chen Y. (2013). Photosensitizer–gold nanorod composite for targeted multimodal therapy. Small.

[71] Shipunova V.O., Belova M.M., Kotelnikova P.A., Shilova O.N., Mirkasymov A.B., Danilova N.V., Komedchikova E.N., Popovtzer R., Deyev S.M., Nikitin M.P. (2022). Photothermal Therapy with HER2-Targeted Silver Nanoparticles Leading to Cancer Remission. Pharmaceutics.

[72] Boca S.C., Potara M., Gabudean A.-M., Juhem A., Baldeck P.L., Astilean S. (2011). Chitosan-coated triangular silver nanoparticles as a novel class of biocompatible, highly effective photothermal transducers for in vitro cancer cell therapy. Cancer Lett..

[73] Thompson E.A., Graham E., MacNeill C.M., Young M., Donati G., Wailes E.M., Jones B.T., Levi-Polyachenko N.H. (2014). Differential response of MCF7, MDA-MB-231, and MCF 10A cells to hyperthermia, silver nanoparticles and silver nanoparticle-induced photothermal therapy. Int. J. Hyperth..

[74] Prateeksha P., Bajpai R., Rao C.V., Upreti D.K., Barik S.K., Singh B.N. (2021). Chrysophanol-Functionalized Silver Nanoparticles for Anti-Adhesive and Anti-Biofouling Coatings to Prevent Urinary Catheter-Associated Infections. ACS Appl. Nano Mater..

[75] Ansari M.A., Kalam A., Al-Sehemi A.G., Alomary M.N., AlYahya S., Aziz M.K., Srivastava S., Alghamdi S., Akhtar S., Almalki H.D. (2021). Counteraction of Biofilm Formation and Antimicrobial Potential of Terminalia catappa Functionalized Silver Nanoparticles against Candida albicans and Multidrug-Resistant Gram-Negative and Gram-Positive Bacteria. Antibiotics.

[76] Khan B., Nawaz M., Hussain R., Price G.J., Farooq Warsi M., Waseem M. (2021). Enhanced antibacterial activity of size-controlled silver and polyethylene glycol functionalized silver nanoparticles. Chem. Pap..

[77] Wei S.-C., Chang L., Huang C.-C., Chang H.-T. (2019). Dual-functional gold nanoparticles with antimicrobial and proangiogenic activities improve the healing of multidrug-resistant bacteria-infected wounds in diabetic mice. Biomater. Sci..

[78] Vial S., Reis R.L., Oliveira J.M. (2017). Recent advances using gold nanoparticles as a promising multimodal tool for tissue engineering and regenerative medicine. Curr. Opin. Solid State Mater. Sci..

[79] Zhang D., Liu D., Zhang J., Fong C., Yang M. (2014). Gold nanoparticles stimulate differentiation and mineralization of primary osteoblasts through the ERK/MAPK signaling pathway. Mater. Sci. Eng. C Mater. Biol. Appl..

[80] Heo D.N., Ko W.-K., Bae M.S., Lee J.B., Lee D.-W., Byun W., Lee C.H., Kim E.-C., Jung B.-Y., Kwon I.K. (2014). Enhanced bone regeneration with a gold nanoparticle–hydrogel complex. J. Mater. Chem. B.

[81] Choi S.Y., Song M.S., Ryu P.D., Lam A.T., Joo S.W., Lee S.Y. (2015). Gold nanoparticles promote osteogenic differentiation in human adipose-derived mesenchymal stem cells through the Wnt/β-catenin signaling pathway. Int. J. Nanomed..

[82] Zhang Y., Wang P., Mao H., Zhang Y., Zheng L., Yu P., Guo Z., Li L., Jiang Q. (2021). PEGylated gold nanoparticles promote osteogenic differentiation in in vitro and in vivo systems. Mater. Des..

[83] Yang E.-J., Lee J., Lee S.-Y., Kim E.-K., Moon Y.-M., Jung Y.O., Park S.-H., Cho M.-L. (2014). EGCG attenuates autoimmune arthritis by inhibition of STAT3 and HIF-1α with Th17/Treg control. PLoS ONE.

[84] Zhu S., Zhu L., Yu J., Wang Y., Peng B. (2019). Anti-osteoclastogenic effect of epigallocatechin gallate-functionalized gold nanoparticles in vitro and in vivo. Int. J. Nanomed..

[85] Alshamrani M. (2022). Broad-Spectrum Theranostics and Biomedical Application of Functionalized Nanomaterials. Polymers.

[86] Báez D.F., Gallardo-Toledo E., Oyarzún M.P., Araya E., Kogan M.J. (2021). The Influence of Size and Chemical Composition of Silver and Gold Nanoparticles on in vivo Toxicity with Potential Applications to Central Nervous System Diseases. Int. J. Nanomed..

[87] Lee J.W., Choi S.-R., Heo J.H. (2021). Simultaneous Stabilization and Functionalization of Gold Nanoparticles via Biomolecule Conjugation: Progress and Perspectives. ACS Appl. Mater. Interfaces.

[88] Barbir R., Jiménez R.R., Martín-Rapún R., Strasser V., Jurašin D.D., Dabelić S., de la Fuente J.M., Vrček I.V. (2021). Interaction of Differently Sized, Shaped, and Functionalized Silver and Gold Nanoparticles with Glycosylated versus Nonglycosylated Transferrin. ACS Appl. Mater. Interfaces.

[89] Foroozandeh P., Aziz A.A. (2018). Insight into Cellular Uptake and Intracellular Trafficking of Nanoparticles. Nanoscale Res. Lett..

[90] Sani A., Cao C., Cui D. (2021). Toxicity of gold nanoparticles (AuNPs): A review. Biochem. Biophys. Rep..

[91] Ko W.-C., Wang S.-J., Hsiao C.-Y., Hung C.-T., Hsu Y.-J., Chang D.-C., Hung C.-F. (2022). Pharmacological Role of Functionalized Gold Nanoparticles in Disease Applications. Molecules.

[92] Ozcicek I., Aysit N., Cakici C., Aydeger A. (2021). The effects of surface functionality and size of gold nanoparticles on neuronal toxicity, apoptosis, ROS production and cellular/suborgan biodistribution. Mater. Sci. Eng. C.

